# Linear Segmented Arc-Shaped Piezoelectric Branch Beam Energy Harvester for Ultra-Low Frequency Vibrations

**DOI:** 10.3390/s23115257

**Published:** 2023-06-01

**Authors:** Iresha Erangani Piyarathna, Ahmed Mostafa Thabet, Mustafa Ucgul, Charles Lemckert, Yee Yan Lim, Zi Sheng Tang

**Affiliations:** 1 Faculty of Science and Engineering, Southern Cross University, East Lismore, NSW 2480, Australia; 2 Fortescue Future Industries Pty Ltd., 160 Lakes Rd, Hazelmere, WA 6055, Australia; 3 Sri Emas International School, Shah Alam 40000, Selangor, Malaysia; 4 College of Engineering, Design and Physical Sciences, Brunel University London, Kingston Lane, Uxbridge UB8 3PH, UK

**Keywords:** arc-shaped branch beam harvester, curved beam, human motion, macro-fibre composite (MFC), piezoelectric energy harvesting

## Abstract

Piezoelectric energy harvesting systems have been drawing the attention of the research community over recent years due to their potential for recharging/replacing batteries embedded in low-power-consuming smart electronic devices and wireless sensor networks. However, conventional linear piezoelectric energy harvesters (PEH) are often not a viable solution in such advanced practices, as they suffer from a narrow operating bandwidth, having a single resonance peak present in the frequency spectrum and very low voltage generation, which limits their ability to function as a standalone energy harvester. Generally, the most common PEH is the conventional cantilever beam harvester (CBH) attached with a piezoelectric patch and a proof mass. This study investigated a novel multimode harvester design named the arc-shaped branch beam harvester (ASBBH), which combined the concepts of the curved beam and branch beam to improve the energy-harvesting capability of PEH in ultra-low-frequency applications, in particular, human motion. The key objectives of the study were to broaden the operating bandwidth and enhance the harvester’s effectiveness in terms of voltage and power generation. The ASBBH was first studied using the finite element method (FEM) to understand the operating bandwidth of the harvester. Then, the ASBBH was experimentally assessed using a mechanical shaker and real-life human motion as excitation sources. It was found that ASBBH achieved six natural frequencies within the ultra-low frequency range (<10 Hz), in comparison with only one natural frequency achieved by CBH within the same frequency range. The proposed design significantly broadened the operating bandwidth, favouring ultra-low-frequency-based human motion applications. In addition, the proposed harvester achieved an average output power of 427 μW at its first resonance frequency under 0.5 g acceleration. The overall results of the study demonstrated that the ASBBH design can achieve a broader operating bandwidth and significantly higher effectiveness, in comparison with CBH.

## 1. Introduction

With the development of the Internet of Things (IoT) and big data, the rapid technological revolution has recently necessitated improved micro-energy harvesting systems to power wireless sensors and communication nodes [1]. This is required to overcome the shortcomings of conventional batteries, including limited life span, capacity, hazardous disposal, and replacement difficulty in several locations. Moreover, in specific practical applications such as implantable medical devices [2] or cardiac pacemakers [3], there arises a need for frequent surgeries to replace batteries that seriously threaten the patient’s health. Thus, long-lasting self-powered smart devices driven by micro energy generation systems possess a clear superiority and desirability compared to their battery-reliant counterparts.

Micro energy harvesters are designed to capture ambient energy from surrounding sources and convert it into electrical energy. Numerous studies have introduced various transduction mechanisms, including electrostatic [4,5], electromagnetic [6,7], triboelectric [8,9], and piezoelectric [4,10], to harvest energy from various ambient sources. With several prominent characteristics comprising high power density, minimal damping, good scalability, and simplicity of design and implementation, the piezoelectric transduction mechanism has certainly established a unique position compared to the other techniques [11]. Additionally, the ability of piezoelectric materials to convert mechanical energy directly into electrical energy has piqued the interest of research communities working with vibrating sources [12], as electrical energy could be generated from various sources including but not limited to wind [10], ocean waves [13], structural actions [14], vehicular [15] and mechanical motion [16] with piezoelectric transduction. These sources are widely used in powering remote sensors for structural health monitoring systems and piezoelectric energy harvesting systems [17,18,19]. However, biological action, particularly human motion, has recently been recognised as a vibration source for piezoelectric energy harvesting, bringing up a wide range of possibilities for developing different human-motion-powered devices [20]. For instance, the ability to make piezoelectric energy harvesters (PEH) on a micro-scale in flexible and stretchable minuscule devices is a big step forward in developing self-powered implanted medical devices.

Conventional linear PEH is a cantilever beam structure bonded with one or two piezoelectric transducers in the form of a unimorph or bimorph harvester, respectively, near the fixed end. This is often preferred by most researchers since the cantilever beam structure can be modified to enhance the performance of the harvester as required [21]. More often, a proof mass may be attached to the free end of the PEH to tune the resonance frequency of the harvester [22]. Pillatsch et al. [23] proposed a PEH that operates under the swing motion of the human arm. The harvester had an eccentric rotating magnetic proof mass and a piezoelectric bimorph with a magnetic tip mass. It was similar to the structure employed in the Seiko kinetic wristwatch. Moving forward, Shukla and Bell [24] invented a PEH consisting of a rotor pendulum with several strikers and a PVDF unimorph as a piezoelectric system to harness power from the waist motion. Using the lower limb motions, such as leg swings, is more beneficial since they can deliver the most mechanical energy as their torques are higher than those of other body parts [25]. Pozzi et al. [26] proposed a wearable knee joint PEH that consisted of seventy-four plectra implanted in a rotating hub with four fixed piezoelectric bimorphs. More recently, Izadgoshasb et al. [27] invented a device to enhance the productivity of PEH using the double pendulum system coupled with repulsive magnetic force. The harvester performed significantly better than most existing designs. However, unlike high-frequency energy sources, working with ultra-low frequency vibration (1–10 Hz) [28] sources such as human motion is still challenging due to the intrinsically high resonance frequencies of most conventional linear PEH systems. Besides, the above-mentioned PEH designs are working on a frequency-up conversion method that contains considerable additional weights, magnets, impact stoppers, etc. So far, studies focusing on ultra-low-frequency vibrations with geometrical optimisation of the design are relatively scarce. 

Conventional PEH is designed to operate at its first resonance frequency within a narrow operating bandwidth. Hence the effectiveness of the harvester becomes significantly poorer in an environment where abundant ambient vibrations are randomly spread over a wider frequency spectrum. PEH with multiple branches is feasible to attain multiple close resonance peaks. Zhang and Hu [29] studied a PEH with multiple branches attached to the main cantilever beam. The design performed better in power density than the conventional PEH since a single piezoelectric patch was used to generate multiple resonance peaks. Further studies on the branch beam concept were conducted by Upadrashta et al. [30], Izadgoshasb et al. [31] and Piyarathna et al. [32]. Even though the devices mentioned above were superior to the conventional PEH, they either carried excessive proof masses for performance improvement, were not satisfying for multi-directional ultra-low frequency vibrations, such as human motion, or had limited performance improvement in an ultra-low frequency range.

Another major drawback of conventional PEHs is they cannot generate adequate electricity for most low-power smart electronic devices [33]. Hence, researchers have studied piezoelectric material improvements [34,35], circuitry developments [36,37,38], and structural and mechanical enhancements to improve power output. This paper focuses on the structural aspects of PEH for power improvement. With time, the structure of the conventional cantilever beam has been modified from a rectangular section to different structures, including triangular [39], trapezoidal [40], spiral [41], zigzag [42], and curved sections [33]. Among these, curved sections have recently been utilised in PEHs. These curved sections experience uniform stress distribution along the vibrating direction compared to commonly used conventional cantilever beam harvesters (CBHs), which helps to boost the effectiveness of the PEHs in terms of voltage, power, and power density [33]. Furthermore, the curved PEH is less affected by the charge redistribution effect due to relatively even stress distribution [33,43,44]. However, standalone curved sections cannot widen the PEH’s operating bandwidth. Hence, developing a PEH that can achieve broader operating bandwidth and effective power output simultaneously is challenging.

This paper presents a novel harvester design, which incorporates the curved beam and branch beam concepts together for ultra-low frequency excitations. As per the authors’ knowledge, this was the first time these two concepts had been combined. One of the key objectives of this study was to propose a harvester capable of achieving a few closer resonance peaks in the ultra-low frequency range (0–10 Hz) [28], i.e., to broaden the operating bandwidth. Having a few more comparable resonance frequencies in the ultra-low frequency range would fit well with most common human motions, from the heartbeat [45], lung motion, and muscular contraction displacements [46] inside the human body to external physical motions, including finger movements [47], walking [48], and running [49], which waste an enormous amount of mechanical energy that can be potentially converted into electrical energy. The other objective was to enhance the effectiveness of the harvester in terms of voltage and power output purely through geometrical optimization without any additional accessories (i.e., no magnet/impact stopper penalty). The design was validated by finite element analysis (FEA) and experimental studies. FEA was conducted to assess the operating bandwidth, while the experimental study was focused on evaluating the voltage and power capability of the proposed design. Experimental tests were conducted using a mechanical shaker and human motion to illustrate ultra-low-frequency energy sources. It is worth noting that this paper is focused on an experimental study to prove the workability of combining two concepts (curved beam and branch beam concept). Hence the theoretical study can be considered for advanced development of the harvester in future research.

## 2. Design of Arc-Shaped Branch Beam Harvester

**Figure 1:**
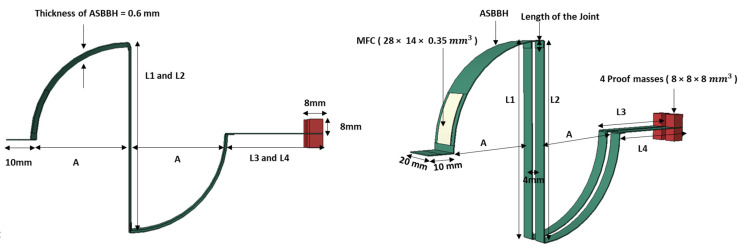
Schematic of proposed ASBBH. (A = 60, L1 = L2 = 120, L3 = L4 = 60).

This study proposed a novel beam design by combining arc-shaped cantilever beam sections with two branch beam sections, as presented in Figure 1. The arc-shaped cantilever beam acts as the main beam component of the design. A 28 mm × 14 mm × 0.350 mm sized macro fibre composite (MFC—M2814-P2) patch operating under bending mode ($d_{31})$ was attached with epoxy near the main beam’s fixed end. Two branches comprising a vertical straight section, arc shape section, and horizontal straight section were attached to the main beam’s free end, allowing a 4 mm gap in between. The proposed arc-shaped branch beam harvester is referred to as ASBBH in the text. A parametric study was conducted to understand the effect of using different beam lengths for ASBBH, presented in Section 3.1.

**Figure 2:**
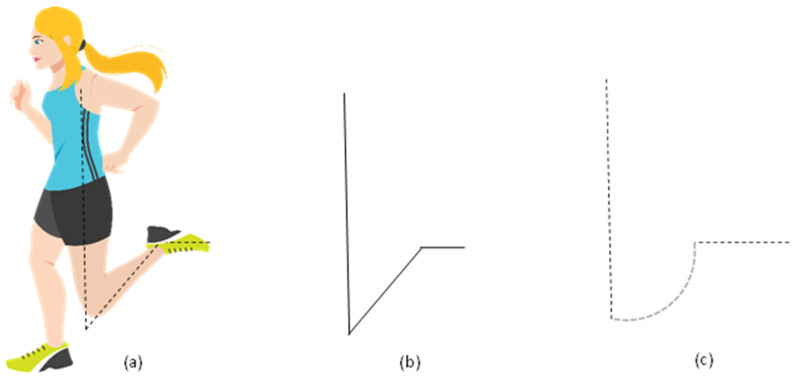
Architecture of the branch beam (**a**) End of stance phase and beginning of swing phase of running motion (**b**) Inspired branch shape (**c**) modified branch shape.

The architecture of the branch beam was inspired by the stance phase and swing phase of running motion, as shown in Figure 2. At this stage, the leg that strikes the ground acts as a shock absorber helping to dissipate the mechanical energy of the body [50]. This allows the bent leg to enter the swing phase [50]. The highest knee flexion (i.e., bending of the knee) is achieved during the end of the stance phase and the start of the swing phase for running motion [51]. Inspired by this phenomenon, the branch beam of the harvester has been designed (Figure 2b) to achieve higher flexion. However, the design was further modified to Figure 2c, as a curved joint section helps to distribute the stress evenly compared to straight sections, aiming to increase the durability of the harvester when it experiences excessive deformations. While working under vibrations, the branch beams induce tensile stress along the beam axis. Following this, the branch beam motion causes flexural deformation in the arc-shaped main beam near the clamped end, which tends to generate strain in the MFC attached to the arc-shaped main beam. In this manner, the excitation tends to amplify twice, initially by the branch beams and then by the flexural deformation of the arc-shaped main beam [52].

**Figure 3:**
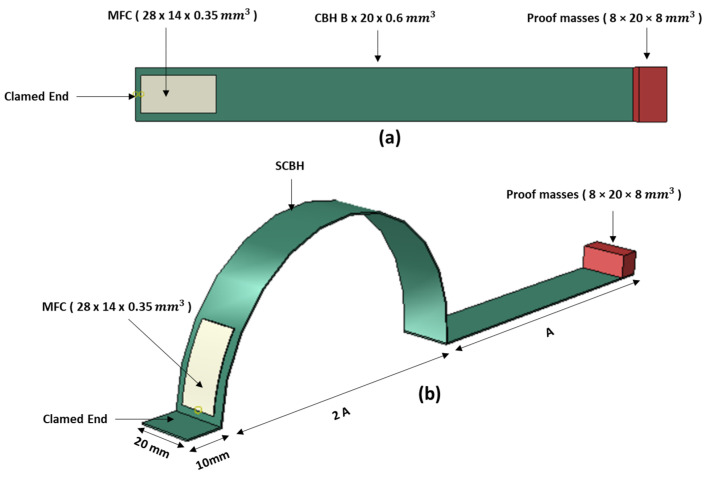
Schematics of PEH designs (**a**) CBH (**b**) SCBH.

Two alternative beam designs (conventional cantilever beam harvester (CBH) (Figure 3a) and segmented curved beam harvester (SCBH) (Figure 3b) were also explored in the FEA to evaluate the operating bandwidth and effectiveness of the proposed device. To achieve a fair comparison, the overall horizontal length of ASBBH was considered as the length of the CBH. The equal volume concept was not considered in this scenario to avoid the length of the CBH becoming excessive. The volume of the other counterpart, SCBH, was kept equivalent to the ASBBH. A proof mass of similar weight was attached to the free end of all three harvester designs. 

The physical parameters of the aluminium beam and steel proof mass are summarised in Table 1. Furthermore, the material properties of the MFC transducers used in this study are summarised in Table 2. These values have been obtained from the Smart Materials Corporation manufacturer’s profile.

## 3. Finite Element Analysis (FEA)

For the proposed ASBBH, FEA was conducted using the commercially available FEA software package SIMULIA ABAQUS to evaluate its mechanical behaviour. The findings of the FEA study will lead the authors to understand the most suitable beam section lengths, the proximity of natural frequencies, the harvester’s operating bandwidth, and the potentiality of voltage and power generation. CBH and SCBH systems were also examined using FEA for comparison purposes.

Three types of simulations were performed in FEA: parametric study, static analysis, and modal analysis. A fixed constraint was applied at one end for all three harvesters, and a proof mass was attached to the free end. The physical properties adopted for the simulation are presented in Table 1 and Table 2. A 20-node quadratic piezoelectric brick element named C3D20RE was used to model the MFC piezoelectric patch attached to each harvester. A 20-node quadratic brick element named C3D20R was used to build the model for the rest of the solid beams and proof masses. Each node possessed three translational degrees of freedom in the polar coordinate system for both elements. The element size chosen was 1 mm after a discretisation and convergence test, which was small enough to produce accurate results without requiring significant processing effort [31].

### 3.1. Geometrical Optimisation of the ASBBH—Parametric Study

The natural frequencies (NFs) and modal characteristics of a harvester can be altered by changing the geometrical parameters, including beam length, width, thickness and tip mass. The parametric study was conducted in this research to understand the effect of using different beam lengths for ASBBH. This helps to determine the optimum beam lengths for the different beam sections to operate the harvester below 10 Hz, preferably with one or more NFs. As such, only the beam lengths were changed, while the beam width, thickness and proof mass were kept constant. However, the values of the fixed parameters were selected as below due to the following reasons.

The width of the piezoelectric transducer (MFC) used in the study was approximately 15 mm; thus, the curved beam width was fixed as 20 mm. The thickness of the selected aerospace-graded aluminium material was 0.6 mm; hence beam thickness was fixed at 0.6 mm throughout the harvester. The minimum gap between the branch beams that allowed the branch beams to vibrate without colliding with each other was 4 mm; hence the gap between the branches was set as 4 mm for the whole branch beam section. The minimum arc length considered in the parametric study was 70 mm, considering the length of the MFC patch (30 mm), clamped edge (10 mm) and length of the joint (5 mm). The upper and lower arc beams were kept with the same arc lengths. Table S1 represents the first three NFs of various design configurations selected in the parametric study. A few of the configurations (arc lengths between (70–120 mm) were not listed in Table S1 for simplicity. All the design parameters were labelled in Figure 1.

The desired configuration of the ASBBH with ultra-low, closer natural frequencies was achieved with a 120 mm arc length. Out of the three designs with the respective arc length, designs 5 and 6 work well for the frequency of human motion. However, to avoid hogging the L3 beam (in design 6) due to its excessive length, design 5 was selected as the preferred configuration for ASBBH. However, it is worth noting that these dimensions of the harvester (ASBBH) were chosen as a proof of concept. With the special characteristics of piezoelectric materials, the device can be scalable to use as enlarged or as a microstructure depending on the real-time application.

### 3.2. Static Analysis

**Figure 4:**
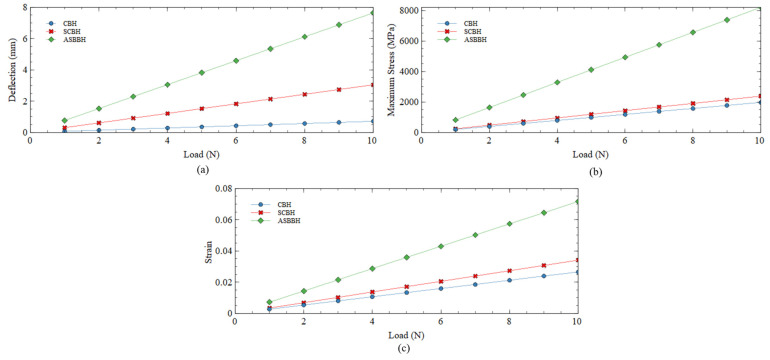
(**a**) Tip deflection (**b**) Highest stress (**c**) Highest strain gained for all three harvesters under different applied loadings.

The operating mechanism of a transducer is a critical factor for any energy harvester design. The MFC transducer used for this study, which works under the d_31_ mode, relies on the strain generated in the cantilever beam. Hence, static analysis was first conducted in ABAQUS for all three designs to elucidate the highest stress/strain gain. During the analysis, a different magnitude of imaginary loads (1–10 N) was applied at the midpoint of the proof mass attached to the tip of each energy harvester. In the case of ASBBH, the loads were applied only to the top two proof masses out of the four, keeping the sum of the loads equal to the load applied to the other two designs at similar conditions. The two masses on the bottom of the branch beam were not added with any loads as it was unrealistic in actual conditions and did not replicate the same conditions as the other two harvesters, CBH and SCBH, respectively. The results were elaborated on tip deflection and highest stress/strain gain and presented in Figure 4.

#### 3.2.1. Tip Deflection

This section discusses the tip (free end) deflection of each harvester against the applied load. The deflection of a cantilever beam is linearly proportional to the stress and strain gained. The deflection is proportional to the stress/strain generation, thus, voltage production.

As per the results presented in Figure 4a, the tip deflection of CBH at all applied loads (1–10 N) remains the lowest. For an instant, the deflection of SCBH and ASBBH was approximately four times and 10 times higher than that of CBH. Moreover, ASBBH achieved about 2.5 times higher deflection than SCBH for all considered cases. This allows us to predict that the achievable stress/strain might follow the same trend in their results. In accordance with the visual observations made during the experiments for voltage generation, the two branches attached to the main beam of ASBBH significantly enhanced the main beam deflection.

#### 3.2.2. Highest Stress and Strain

The main beam’s highest stress/strain generation is crucial for energy generation by the MFC patch. Figure 4b,c present the highest calculated stress/strain results under different loadings (1–10 N). The highest stress/strain of CBH was generated near the fixed end of the harvester. However, it was noted that the aforementioned stress/strain generation of CBH was the lowest among all three energy harvesters. Compared to the stress/strain generation of CBH, SCBH slightly improved its performance by approximately 1.2 times and 1.3 times, respectively.

In contrast, the proposed ASBBH yielded the highest stress and strain, which were comparatively higher than the other two designs. The stress generation of ASBBH was approximately 3.4 times and four times higher than SCBH and CBH, respectively. Conversely, the strain generation of ASBBH was approximately two times and 2.7 times higher than SCBH and CBH, respectively. Possible reasons for this significant improvement of ASBBH could be the higher tip deflection and the reduced stiffness due to the compact structure. These results emphasised that the proposed ASBBH could potentially generate higher voltage and power when dynamically excited. To sustain the higher stress and strain experienced during the vibration, the ASBBH design is recommended to fabricate with aerospace-graded aluminium 2024. As per the manufacturer’s profile, aerospace-graded Aluminium 2024 has high strength and excellent fatigue resistance compared to aluminium 6061. However, future studies are recommended to focus on assessing the long-term reliability of the ASBBH.

### 3.3. Modal Analysis and Mode Shapes

#### 3.3.1. Modal Analysis

**Figure 5:**
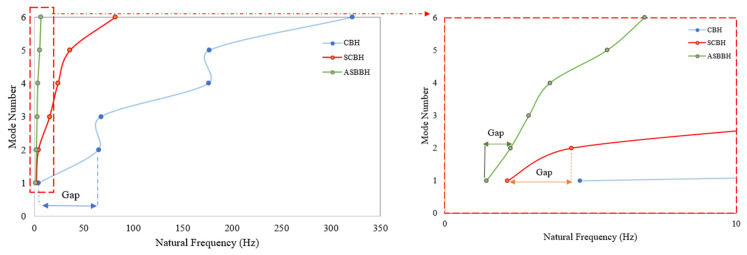
Natural frequencies and their gap between each natural frequency for all three harvesters.

To identify the first six natural frequencies and mode shapes, three-dimensional (3D) structures for ASBBH, CBH, and SCBH were investigated in SUMULIA ABAQUS. To do so, a linear perturbation analysis was conducted. The software used the following Equation (1) to solve the eigenvalue problem in this analysis, where ω was the frequency of the system while M represented the mass matrix, *φ* denoted the mode shape, and *K* symbolised the stiffness of the system. The natural frequencies obtained through FEA are presented in Table 3 and Figure 5.

(1) $$-\omega^{2}M\varphi +K\;\varphi =0$$

As per the results, the first six natural frequencies of CBH spread over a frequency range of 4.6 Hz to 322 Hz. In addition, CBH had only one natural frequency in the desired ultra-low frequency spectrum (<10 Hz). The gap between the first and second natural frequency was approximately 60 Hz, an inherent expanse. Compared to CBH, SCBH has two natural frequencies in the ultra-low range. Further, the natural frequencies’ spread had been lowered from 2.15 Hz to 82.22 Hz. In this case, the gap between the first and sixth natural frequencies was approximately 80 Hz, reflecting that the CBH had a narrower operating bandwidth than SCBH.

In contrast, the proposed ASBBH exhibited significant enhancement compared to CBH and SCBH. For instance, ASBBH achieved six natural frequencies in the desired frequency spectrum (<10 Hz), a six-fold and three-fold improvement over CBH and SCBH, respectively. It is well-known that the PEH could generate the highest output power when the system, particularly the cantilever beam, excites at its natural frequency [21]. With six natural frequencies in the ultra-low range, ASBBH facilitates the potential to generate high power output compared to other counterparts. In addition, the gap between each natural frequency increment (e.g., NF1, NF2, NF3, NF5 and NF6) did not exceed 1.96 Hz. Thus, most of the frequencies relying on the band gap have the potential to generate considerable power output since they are much closer to natural frequencies. This statement could be further demonstrated in the experimental stage. Moreover, this reduction in gap leads to a lower spread over six natural frequencies of ASBBH within 1.43 Hz to 6.85 Hz, increasing the operating bandwidth of the harvester, solving one of the major issues of CBH.

#### 3.3.2. Mode Shapes of ASBBH

**Figure 6:**
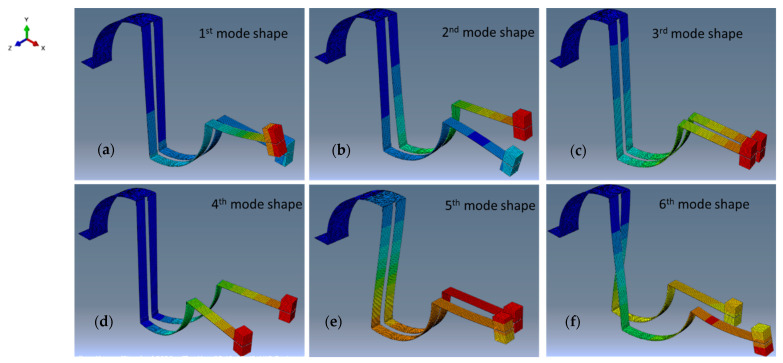
First six mode shapes obtained of ASBBH.

Figure 6 demonstrates that the branches of ASBBH have motions in three-dimensional space. The X-direction is along the harvester length, the Z-direction is along the harvester width, and branches fixed to the curved beam move in the Y-direction. Hence, the proposed ASBBH demonstrates the capability of harvesting energy through multi-directional vibrations. Moreover, the authors visually witnessed this motion of the branches while conducting the experiments. Generally, this motion would be limited for a conventional cantilever beam harvester, having the beam bending along the Y-direction only. Branch beam harvesters proposed in previous studies [30,31] also have a limited motion with beam bending along the Y-direction.

In addition, the mode shapes presented in Figure 6 further demonstrate that the first, second, fifth and sixth mode shapes (Figure 6a, Figure 6b, Figure 6e and Figure 6f, respectively) were bending dominant as the beam segments experienced deflection in their transverse direction. Having few bending-dominant modes in ASBBH would be beneficial for electricity generation because the D_31_ operating mode of MFC used in the proposed harvesters tends to convert strain into electricity, mainly due to the bending in the Y–direction. The third and fourth modes were mainly categorised as torsion-dominant since the beam segments illustrate a rotation around their respective axis. In the early stage of PEH systems, the torsion-dominant mode was frequently averted, believing opposing signs of stress and subsequent charge cancellation in a flat piezoelectric patch. However, this was later proven false, as in the torsion-dominant mode coupling both bending and torsional modes was found to be comparatively effective [43].

## 4. Experimental Study

To further validate the FEA studies, the performance of the proposed ASBBH was investigated with a series of experimental tests conducted in two stages. The ASBBH was excited with controlled constant impulse acceleration [27] and stable low-frequency vibrations generated by a mechanical shaker at the first stage. The constant impact acceleration was generated to emulate typical human motions [27]. In the second stage, the harvester was excited by human motions, including walking, jogging, and running, to illustrate the suitability of ASBBH under realistic complex ultra-low frequency vibrations. The experimental results were also evaluated regarding operating bandwidth, output voltage, output power, idle time, and average power.

The proposed ASBBH was fabricated per the detailed design presented in Section 2. Aero-graded aluminium was used to fabricate the beam sections while four steel proof masses weighing 4 g were attached at the free end of the two branch beams. A D_31_ type MFC (MFC—M2814P2) transducer was attached near the fixed end of the main beam with two epoxy parts. The physical properties of all aero-graded aluminium, MFC and steel are presented in Table 1 and Table 2. It is worth noting that the dimensions of the harvester (ASBBH) were chosen as a proof of concept, and with the special characteristics of piezoelectric materials, the device can be scalable to use as microstructures for real-time applications.

### 4.1. Stage 1: Shaker Test

In a controlled environment, the ASBBH was excited by a mechanical shaker under constant impulse acceleration [27]. One end of the proposed harvester was clamped to an electrodynamic shaker (APS-113, APS Dynamics, Inc., Dresden, Germany) to experience ultra-low frequency vibrations. To operate the mechanical shaker, the required frequency input was fed by the function generator (Agilent 33210A, Santa Clara, CA, USA) while power was controlled by a power amplifier (APS125, APS Dynamics, Dresden, Germany). At the base of the ASBBH, an accelerometer (Dytran 3305A2, 0.3 to 5000 Hz, ±5%, Chatsworth, CA, USA) was fixed to sense the ongoing impulse acceleration. Output voltage and acceleration were measured by NI DAQ modules, NI 9229, and NI 9234, respectively, and recorded using Signal Express software. The power output (P) was calculated using harvested voltage (V) under open circuit conditions. Since the experiments were conducted under open circuit conditions, nearly infinity, they require a considerably higher load resistance to calculate the power output. In addition, the finite input impedance of the NI DAQ module (NI 9229) was 1 MΩ. Hence, considering both facts, to calculate the power output of ASBBH, 1 MΩ of load resistance was assumed [21]. The expressions of calculating the power output and average power output are given in Equations (2)–(4), respectively.

(2) $$P=V^{2}/R_{load}$$

(3) $$P_{average}=V_{rms}^{2}/R_{load}$$

(4) $$V_{rms}=V/\sqrt{2}$$

**Figure 7:**
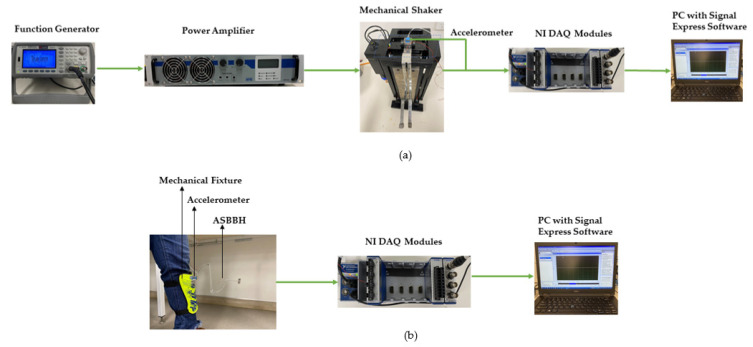
(**a**) Schematic of experimental setup, (**b**) Schematic of experimental setup for the human motion test.

During the design stage of the ASBBH, the clamped edge was kept a little away from the curved main beam by purposely having an additional horizontal 20 × 10 × 0.6 mm^3^ aero-graded aluminium piece (see Figure 1). This was adopted in the design mainly to protect the MFC patch, as the ultra-low frequencies experience higher amplitudes while vibrating. The schematic of the mechanical shaker test is presented in Figure 7a. The proposed ASBBH was tested throughout a frequency sweep of 1–6 Hz at a step of 0.25 Hz. Each frequency was sequentially assessed under two accelerations (i.e., 0.5 g and 1 g).

### 4.2. Stage 2: Human Motion Test

The harvester was analysed under human motion as the sole vibration source in stage two. In common practice, a human motion could be elucidated as a complicated live motion occurring in various directions with different amplitudes and accelerations, depending upon the intention and source of movement. Most often, the frequency of human motion belongs to the ultra-low frequency range with high amplitudes [53]. Common human motions occur under different frequency ranges, as explained in Table 4. The corresponding accelerations for these motions differ from one person to another due to various reasons, including lifestyle, weight, height, age, medical conditions, and many more [54,55,56]. For this study, common, steady, and repetitive human motions, including walking, jogging, and running, were selected as excitation sources. Human leg swing was focused on the above motions. A customised mechanical fixture was fabricated to attach ASBBH to the leg, as shown in Figure 7b. The test subject was a 56 kg weight and 150 cm tall female, and the prototype (i.e., mechanical fixture carrying the harvester) was affixed to the bony landmark location [57]. It is worth stating that attaching the prototype to the bony landmark location helps to lessen the relative motion between the prototype and the bone, reducing the damping effect caused by the skin [12]. Further, the harvester was tightened to the mechanical fixture with screws to preserve the fixed-end condition.

An accelerometer was mounted at the base of the main beam to capture the acceleration caused by walking, jogging, and running motions. In general, the acceleration caused by different subjects could depend on some or all of their height, weight, and the habit of motion pattern. For all the above-mentioned motion types, the outcome is governed by vertical acceleration for this study, as the impact caused during a foot strike is predominantly induced by vertical acceleration [58,59,60,61]. To acquire a fair experimental condition, each test for walking, jogging, and running motions was conducted at least three times under constant acceleration of 0.3 g, 0.6 g and 0.8 g, respectively. To attain these accelerations in a constant state, the test subject was required to walk/jog/run continuously, not less than 5 min before data acquisition was commenced. Corresponding frequencies continuously measured across 10 s for the same motions were identified as 1 Hz, 1.5 Hz and 1.7 Hz. As with the shaker test, output voltage and acceleration were recorded using NI DAQ modules. Later, the generated power was calculated using Equation (2) under open circuit conditions.

**Table 4:** Frequency ranges for common human motions.

Reference	Human Motion Type	Frequency Range of Interest (Hz)
Kumar, Anuruddh, et al. [45]	Hear beat	1–1.5
Dong, Lin, et al. [46]	Lung motion	0.2–0.5
Kim, Jae Woo, et al. [47]	Finger-triggering	0.5–5
Maharjan, Pukar, et al. [62]	Hand shake	0.5–6
Cai, Mingjing, et al. [48]	Walking	0.5–2
Cavagna, G. A., et al. [49]	Running	1–5

## 5. Results and Discussion

### 5.1. Shaker Test

#### 5.1.1. Output Voltage

**Figure 8:**
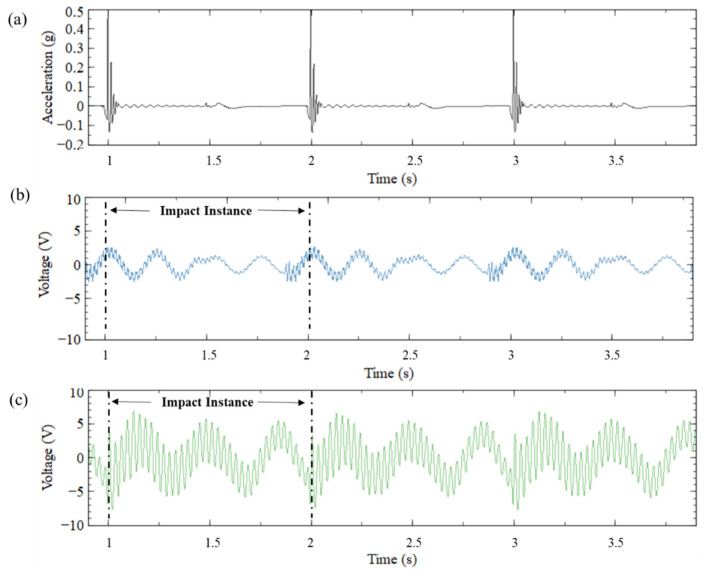

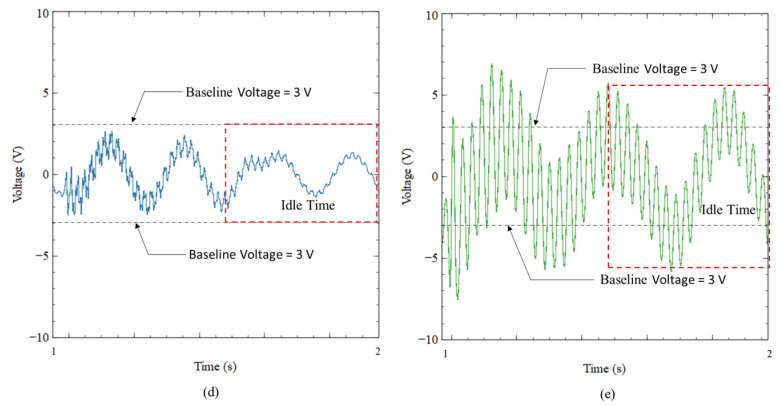
(**a**) Base acceleration (**b**) Voltage output of CBH (**c**) Voltage output of ASBBH (**d**) Idle time for CBH and (**e**) Idle time for ASBBH under 1 Hz and 0.5 g acceleration.

The first set of experiments was conducted for the ASBBH for a frequency sweep of 1–6 Hz, with steps of 0.25 Hz, under 0.5 g and 1 g impulse acceleration. According to the evaluations made by the FEA study, it was noted that the ASBBH performs better than SCBH. Hence, the experiments were focused only on ASBBH, and SCBH was not considered for experiments. However, for a base comparison, shaker tests were conducted for CBH with the same frequency sweep under the same dynamic conditions. CBH was selected instead of SCBH as it is the conventional piezoelectric energy harvester in general use. Figure 8 illustrates the output voltage achieved by CBH (Figure 8b) and ASBBH (Figure 8c) under 0.5 g acceleration, considering three motion cycles. The peak output voltage obtained for CBH and ASBBH under 0.5 g acceleration was approximately 3 V and 8 V, respectively. This is a remarkable 2.7 times higher voltage improvement of ASBBH compared to CBH. The results for 1 g acceleration showed a similar trend with different voltage magnitudes, hence they have been omitted from the graphical representations of waveforms for simplicity.

#### 5.1.2. Idle Time

The results presented in Figure 8b,c can be further explained in terms of idle time which is a crucial indicator for any energy harvesting device. In this study, the period that generates relatively low voltage compared to the peak voltage is referred to as the idle time of the harvester. Normally, a higher damping could be seen within the idle time. In other words, when the idle time is longer, fewer spikes (i.e., voltage peaks) arbitrate in a motion cycle (i.e., one second), resulting in lower voltage and output power. Hence, idle time is an important parameter to assess the effectiveness of the PEH.

Figure 8d and Figure 8e help to visually illustrate the idle time of CBH and ASBBH in the motion cycle separately. The peak voltage obtained by CBH was nearly 3 V, representing a single impact given at the base of CBH by the mechanical shaker. Hence, to elaborate on the idle time of each harvester, the aforementioned peak voltage of CBH is considered the baseline voltage (i.e., 3 V). The peak voltage of CBH, which is generated at the impact instance, then gradually decreases due to the effect of damping. As per Figure 8d, this damping effect lasts at least half of the motion cycle (at least 0.5 s) until the next impact instance occurs. Compared to the CBH, the ASBBH could generate the baseline voltage output throughout the motion cycle, highlighting the reduction in idle time from 0.5 s to 0 s. If the same baseline voltage is considered for the ASBBH, the damping of the harvester is nearly 0% (no idle time) compared to CBH during the whole motion cycle.

#### 5.1.3. Operating Bandwidth

**Figure 9:**
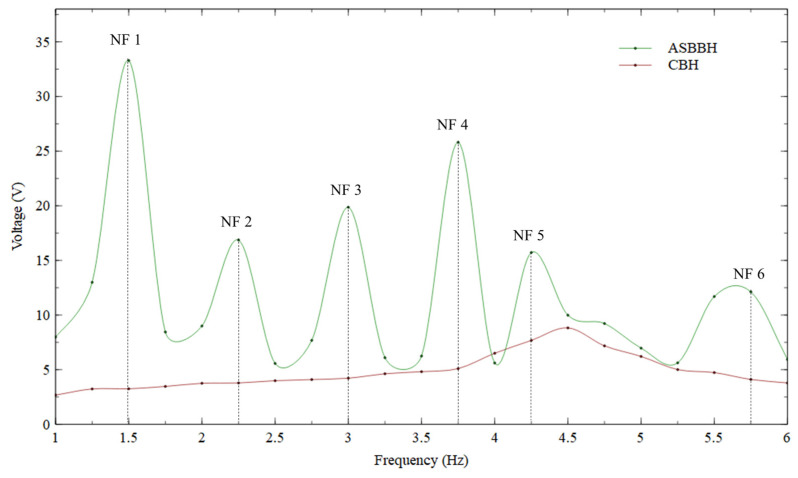
Peak voltage obtained by ASBBH and CBH under 0.5 g acceleration.

The operating bandwidth of a PEH is another vital factor in evaluating its performance. The effectiveness of the PEH could potentially enhance by widening the operating bandwidth. Thus, testing the frequency sweep of 1–6 Hz was essential to prove the results achieved by FEA analysis. Figure 9 illustrates the peak output voltage obtained for both CBH and ASBBH under 0.5 g acceleration across the above frequency sweep (1–6 Hz) at a step of 0.25 Hz.

As per the data illustrated by Figure 9, there were six voltage peaks identified in the frequency range of interest (1–6 Hz) which are recognised as the natural frequencies of the proposed ASBBH. These results agreed well with the natural frequencies predicted under FEA modal analysis. For easy reference, the FEA natural frequencies and experimental natural frequencies are listed in Table 5. The maximum discrepancy between the two studies was limited to 1.5 Hz. This could be due to minor human errors during the fabrication process. According to the results presented in Table 3 for FEA modal analysis, CBH could generate only one natural frequency within the frequency of interest, which agrees well with the results illustrated in Figure 9. Thus, the experimental tests’ results further prove the significant widening in the operating bandwidth of the ASBBH, having six natural frequencies in the ultra-low frequency spectrum compared to the CBH.

#### 5.1.4. Voltage Generation at Different Natural Frequencies of ASBBH

This section discusses the effectiveness of the achieved natural frequencies of the ASBBH in terms of voltage generation under 0.5 g acceleration. The results for 1 g acceleration showed a similar trend with different voltage magnitudes, and hence have been omitted from the discussion for simplicity.

As presented in Figure 9, the peak voltage output of 34 V was acquired for the first natural frequency. This is a significant improvement for a low impact, such as 0.5 g acceleration. Previously under Section 3.3.2. in the FEA mode shape analysis, it was identified that the first natural frequency of the ASBBH was bending dominant. Hence, it was preferable to excite the ASBBH at its fundamental frequency to obtain the maximum electrical response. However, this does not indicate that the voltage output at the rest of the natural frequencies was inadequate because all the other natural frequencies could successfully attain a minimum of 10 V throughout one motion cycle under a low acceleration of 0.5 g. The peak voltage obtained for the second natural frequency was approximately 17 V. If the peak voltage of the first natural frequency (34 V) is considered as the threshold for comparison, the second natural frequency could achieve only 50% of the threshold voltage at each motion cycle.

On the other hand, the third natural frequency could generate approximately 20 V of peak voltage, nearly 60% of the threshold voltage. A slight improvement was noticed in the peak voltage at the fourth natural frequency (26 V), which was 75% of the threshold voltage. The fifth and sixth natural frequencies could obtain 47% and 35% of threshold voltage, respectively. As stated in the FEA mode shape analysis, the third and fourth natural frequencies could be categorised under torsion-dominant modes. According to the above experimental results, they were more effective than the bending-dominant modes, excluding the fundamental bending mode. Thus, the coupling of both bending and torsional motions was comparatively effective. This phenomenon was recently proved in the literature, and the present study’s results further verify the findings of [43].

#### 5.1.5. Harvested Power

**Figure 10:**
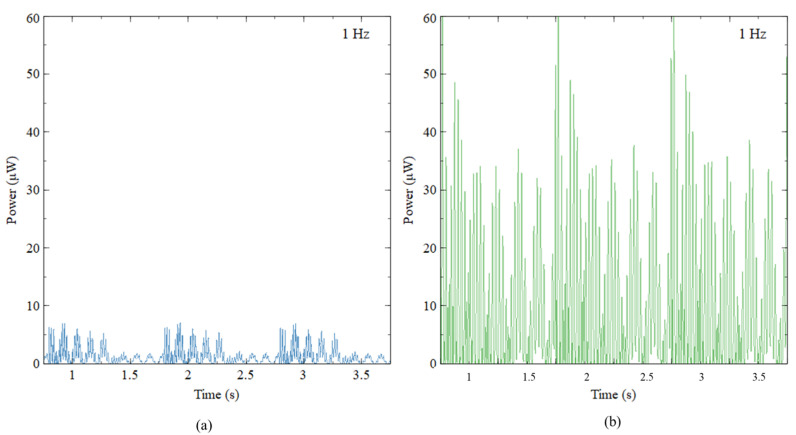
Power generated by (**a**) CBH and (**b**) ASBBH under 1 Hz and 0.5 g acceleration.

The output power harvested by CBH and ASBBH under 0.5 g acceleration was computed and presented in Figure 10. The power was calculated using Equation (2). Since the experiments were conducted under open circuit conditions, nearly infinity, they require a considerably higher load resistance to calculate the power output. In addition, the finite input impedance of the NI DAQ module (NI 9229) was 1 MΩ. Hence, considering both facts, to calculate the power output of ASBBH, 1 MΩ of load resistance was assumed [21,30,31]. It was observed that the ASBBH generated a maximum power of 60 μW while the CBH harvested a maximum power of 7 μW under 0.5 g acceleration. This was an improvement of nearly eight times the power for the ASBBH compared with the CBH. As with the voltage generation of the CBH, power generated by the CBH during each motion cycle decreases rapidly due to higher damping. Conversely, the ASBBH managed to generate power ranging from 10–60 μW throughout the motion cycle. Thus, the ASBBH was more competent in power generation compared to the CBH. For future research, using different resistors to calculate the power generation of ASBBH to identify the harvester performance under different loading conditions is recommended.

**Figure 11:**
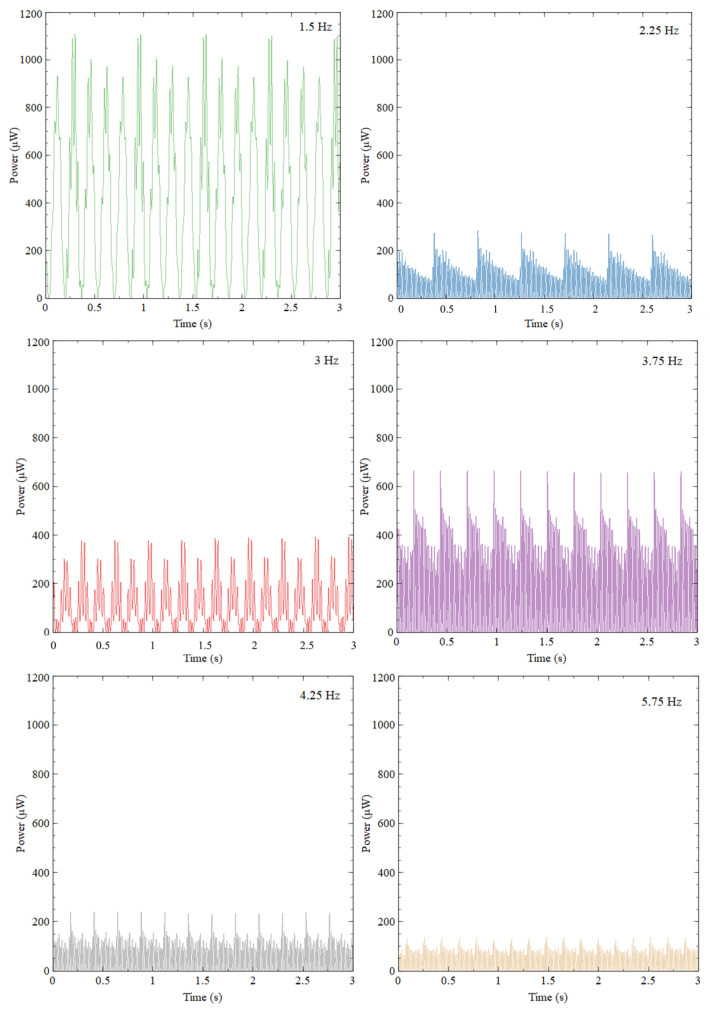
Output power of ASBBH when excited under first six natural frequencies under 0.5 g acceleration.

Next, harvested power for the first six natural frequencies was calculated to further evaluate the proposed design’s productivity. Figure 11 shows the output power collected for the first six natural frequencies under 0.5 g acceleration for three seconds. Similarly, to the voltage output results, 1100 μW of peak power output was collected for 1.5 Hz. The second-best performer was the fourth natural frequency (3.75 Hz), having 650 μW of peak power output. Even though the magnitude of the power obtained for the fourth natural frequency was nearly half the first natural frequency, it could secure a continuous power output of 320 μW throughout the period. The second and third natural frequencies could generate a maximum of 300 μW and 400 μW, respectively. In the second natural frequency, the power between each motion cycle dissipated rapidly due to damping after each impact. The harvester still provided a minimum of 100 μW continuously during each cycle. The worst-performing natural frequencies were the fifth and sixth. However, they could still generate a minimum of 60 μW power throughout the period.

**Figure 12:**
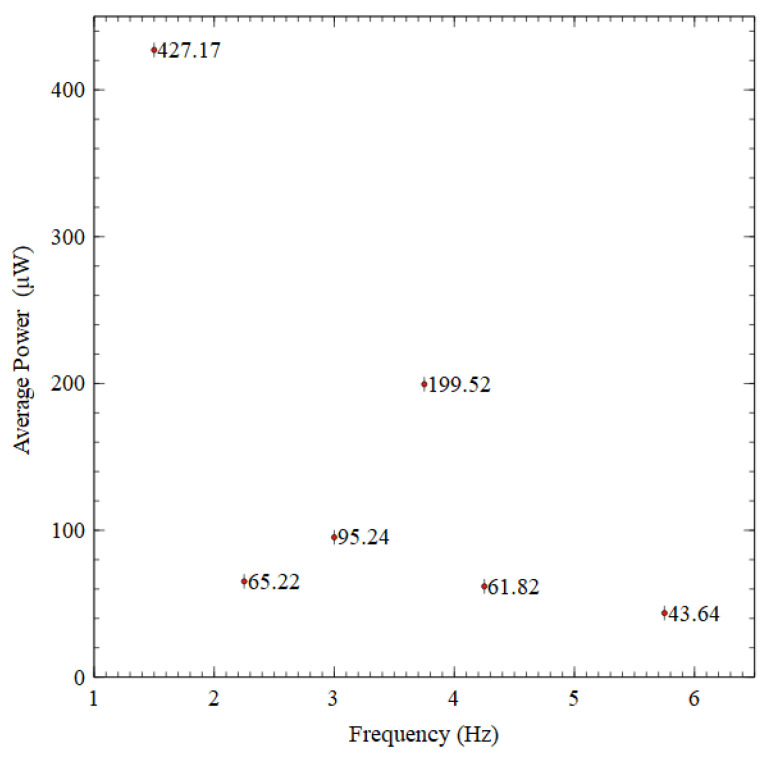
Average output power generated by ASBBH at first six natural frequencies.

To further demonstrate the effectiveness of the proposed design, the average power output (Equation (3)) generated at each natural frequency was computed and illustrated in Figure 12. The power generated within one second was considered for the average power calculations. As expected, the least average power of approximately 44 μW was acquired at the sixth natural frequency. If this is considered the average baseline power (44 μW), the average power generated at first natural frequency was approximately 10 times greater than the average baseline power. The second and fifth natural frequencies could generate about 1.5 times the baseline average power. This was not a significant improvement compared to the baseline average power output. That could be due to the two natural frequencies’ higher damping and lower voltage generation. However, the third and fourth natural frequencies performed better, achieving approximately 2 and 4.5 times the average baseline power.

### 5.2. Human Motion

#### 5.2.1. Output Voltage

This study was conducted to propose an efficient PEH design that could work in ultra-low frequency vibrations such as human motion. Thus, to evaluate the performance of the proposed ASBBH for practical applications, experiments were conducted for three different human motions, including walking (1 Hz), jogging (1.5 Hz) and running (1.7 Hz) and compared against the CBH under similar conditions. At each motion, impact occurs when the heel collides with the ground. Tests were conducted under 0.3 g, 0.6 g and 0.8 g accelerations for walking, jogging and running, respectively. The voltage and power generated during each motion were recorded, analysed, and discussed herein.

**Figure 13:**
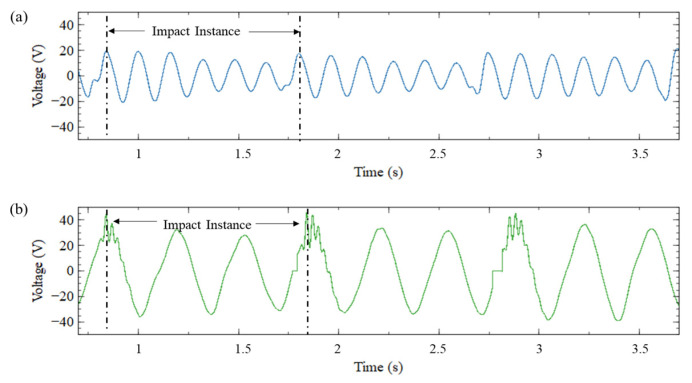

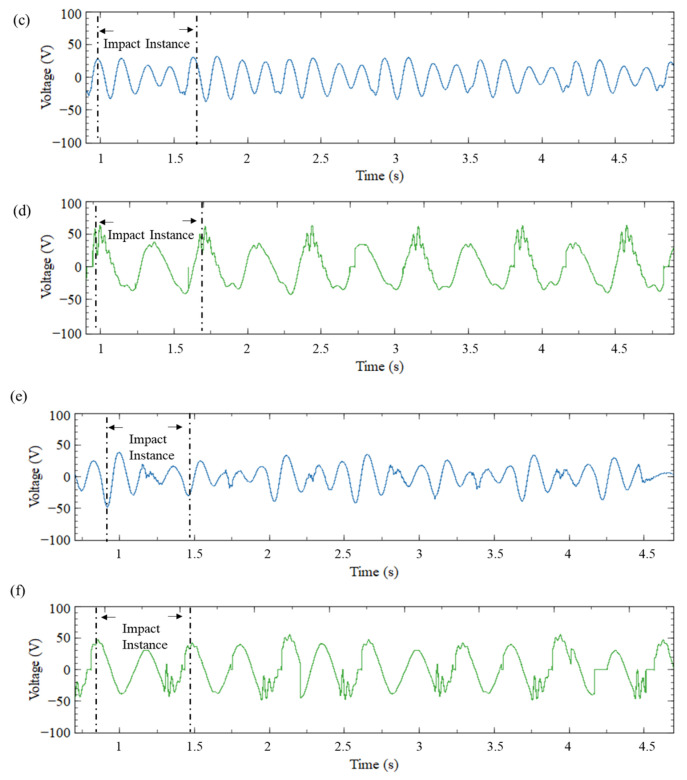
Output voltage generated by walking motion (1 Hz) (**a**) CBH and (**b**) ASBBH, jogging (1.5 Hz) (**c**) CBH (**d**) ASBBH and running (1.7 Hz) (**e**) CBH (**f**) ASBBH.

Figure 13a and Figure 13b presents the voltage generated during human walking by the CBH and ASBBH respectively. The peak voltage obtained by the ASBBH and CBH for walking motion (1 Hz) was approximately 45 V and 20 V, respectively. This was a remarkable improvement gained by the ASBBH compared to the CBH, with more than two times higher peak voltage which agrees with the shaker test results in Figure 8.

Similarly, during jogging (1.5 Hz) and running (1.7 Hz) motions, presented in Figure 13d and Figure 13f, the peak voltages of 65 V and 50 V, respectively, were achieved by the ASBBH. In similar conditions, the peak voltages that the CBH generated were 35 V (Figure 13c) and 40 V (Figure 13e) for jogging and running motions, respectively. This was an improvement of nearly two times and 1.25 times of the ASBBH compared to the CBH performance.

Results obtained for the shaker test could not be compared with the human motion result, as human motion is known to be a complex vibration while the shaker test was conducted under stable vibration conditions. However, irrespective of the different base accelerations, the highest peak voltage was acquired for jogging (1.5 Hz) motion which was identified as the first natural frequency of the ASBBH. Therefore, the proposed ASBBH could potentially harness high power during jogging motion when compared to the other two motion types, walking, and running.

#### 5.2.2. Harvested Power

This section discusses the power generated by CBH and ASBBH using human motion under different motion types. Resembling the open circuit conditions, 1 MΩ resistance was used for the computation with Equation (2).

**Figure 14:**
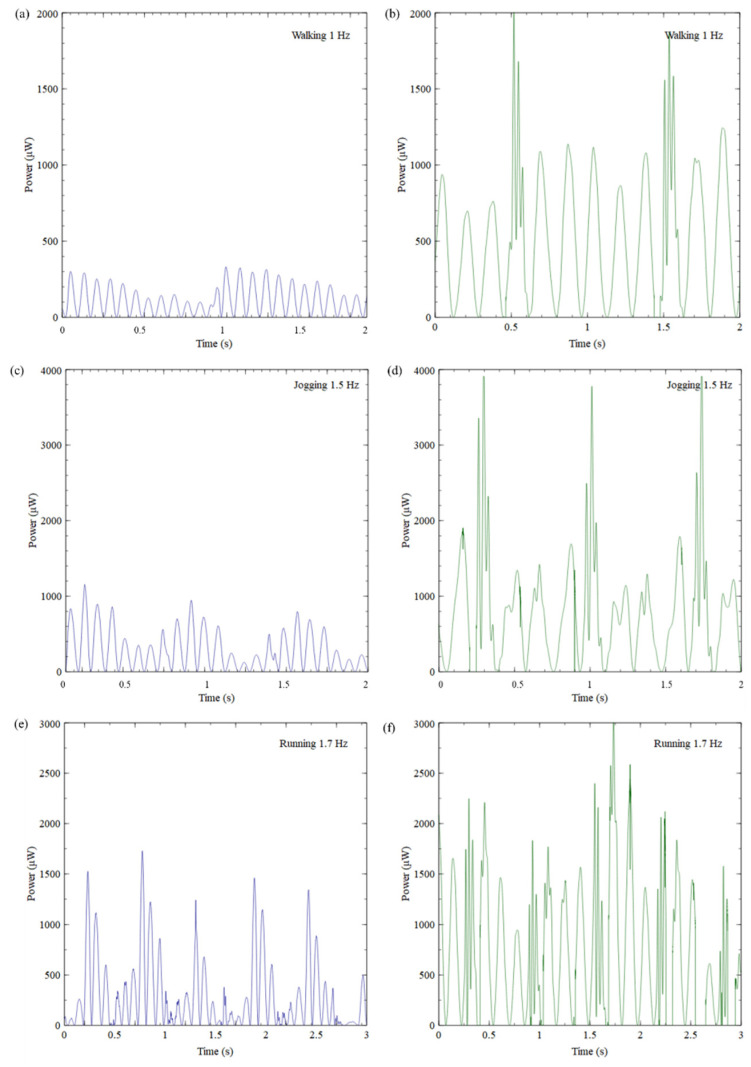

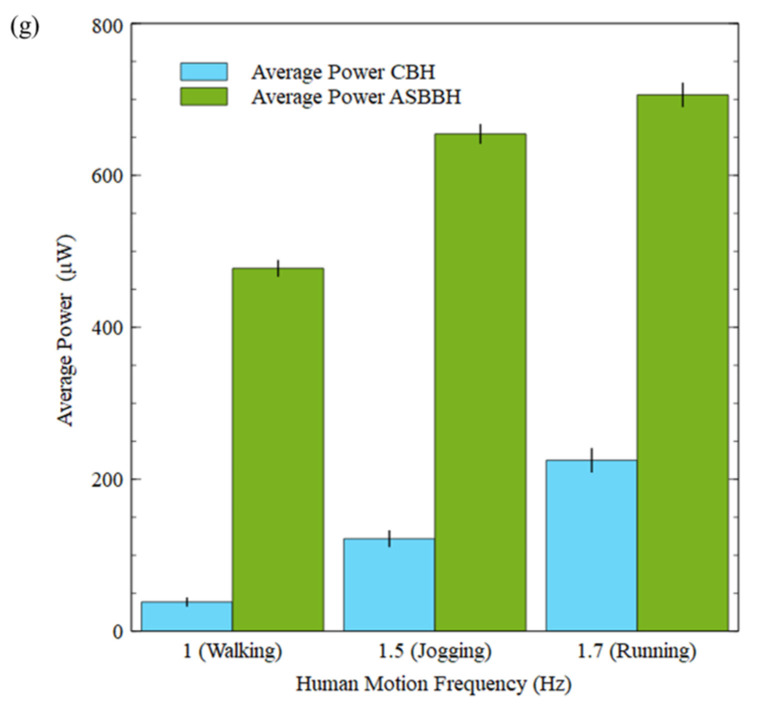
Power harvested by (**a**) CBH and (**b**) ASBBH during Walking motion (1 Hz), (**c**) CBH and (**d**) ASBBH during Jogging motion (1.5 Hz), (**e**) CBH and (**f**) ASBBH during Running motion (1.7 Hz), (**g**) Average Power harvested by CBH and ASBBH during different motion types.

The power harvested by the CBH and ASBBH during walking motion is presented in Figure 14a and Figure 14b, respectively. It was observed that the CBH generated a maximum of 300 μW power while the ASBBH generated nearly 2000 μW of maximum power within one motion cycle. This maximum power (i.e., peak power) was attained during the impact. Similarly, to the previous scenarios (shaker test voltage/power output and human motion voltage output), the power generated by the CBH during walking motion rapidly dissipated due to damping. In contrast, the ASBBH achieved power ranging from 500–1000 μW during the rest of the motion cycle, which was more than the maximum power obtained by the CBH. During jogging motion, the CBH (Figure 14c) generated a maximum of 1000 μW. On the other hand, jogging was the best-performing motion for the ASBBH, where it generated a maximum power output of 4000 μW as presented in Figure 14d, which was four times higher than the maximum power output of the CBH. It is worth noting that during the rest of the time in the motion cycle, the ASBBH obtained a minimum of 1000 μW, apparently the maximum power output of the CBH. Figure 14e and Figure 14f present the power output generated by the CBH and ASBBH in turn during the running motion. The CBH harvested maximum power of 1100 μW while a maximum of 2000 μW was generated by the ASBBH. This was nearly two times higher power generation by the ASBBH compared to the CBH. Unlike walking and jogging motions, several power peaks were visible in the running as more impacts were made while running.

#### 5.2.3. Average Output Power

Next, the average output power generation during each motion type was studied to further understand the effectiveness of the ASBBH compared to CBH. Figure 14g expresses the average power production of the ASBBH and CBH under different human motions during one motion cycle. The average power generation of the CBH and ASBBH gradually increased from walking to running motion. The CBH harvested average output power of 40 μW, 120 μW and 225 μW while walking, jogging, and running motions, respectively. While walking, the ASBBH attained 475 μW of average output power, nearly two times higher than the average output power generated by the CBH during running. The ASBBH generated 650 μW of average output power during jogging motion. The ASBBH’s highest average power of 700 μW was gained during a running motion. This could be mainly due to the running motion’s higher impact (1.7 Hz) and greater number of impacts than walking and jogging motions. Comparatively, the average power of the ASBBH during jogging motion was 50 μW less than the running motion’s output (650 μW). In summary, the ASBBH harvested significantly higher average output power for all three motion types than the CBH, emphasising the favourable design improvements of the ASBBH for piezoelectric energy harvesting using human motion excitations.

#### 5.2.4. Performance Comparison with Existing Designs

A performance comparison with the existing designs in the literature was conducted as per Table 6. Average output power and the number of natural frequencies (<10 Hz) obtained by each harvester design were considered factors for the evaluation. The comparison emphasised that the proposed design works well in ultra-low frequency vibrations compared to the existing designs. The results obtained in this research confirm that the ASBBH has the ability to power low-power consuming wireless sensor nodes (WSNs) (<100 μW) successfully with the aid of a proper rectifier circuit [63,64,65] to stabilize, rectify, and amplify the generated voltage using ultra-low frequency vibrations.

## 6. Conclusions

This study introduces a novel multimode PEH design combining two design aspects, i.e., curved structure and branch beams, to improve PEH’s energy production potential from ultra-low-frequency vibration sources, including human motion. A numerical study using ABAQUS FEA software, and a series of experiments were conducted to examine the proposed ASBBH’s performance in operating bandwidth, voltage generation, and power generation. The numerical study was mainly undertaken to study the operating bandwidth of the harvester, while the experiment series was focused on voltage/power generation and suitability of the harvester in human motion applications. The mechanical shaker and human motion tests (walking, jogging, and running motions) were conducted in the experiments. Its performance was then compared against traditional PEH, i.e., CBH.

Six closer resonance peaks were obtained by the proposed ASBBH within the ultra-low frequency range of 1–10 Hz, while only one resonance frequency was obtained by CBH, emphasising a significant improvement in the operating bandwidth of the harvester. The mechanical shaker test proved that the ASBBH could generate the highest output voltage of 34 V and power of 1100 μW at its first resonance frequency. The remaining five natural frequencies also performed well with regard to voltage and power output. Out of the six natural frequencies, two natural frequencies were operating under torsion-dominant modes. The experimental results showed they were more effective than the bending-dominant modes, excluding the fundamental bending mode. Thus, the coupling of both bending and torsional motions was comparatively effective. In the human test series, a peak output voltage of 65 V and power of 4000 μW was recorded for jogging motion (1.5 Hz), which was identified as the first natural frequency of the ASBBH, irrespective of the different base accelerations. These improvements are significant for a simple harvester design such as ASBBH, as it works without additional penalties but with purely optimised structural parameters of the energy harvester. It is worth noting that the geometrical parameters of the harvester were chosen as a proof of concept, and the harvester can be manufactured as a miniaturised apparatus as required for practical human motion applications.

Combined with a suitable rectifier circuit for power management, the ASBBH could be utilised as a standalone energy provider for low-power-consuming devices such as medical implants and WSNs. For future work, attempts should be made to develop a comprehensive theoretical model to elaborate the working principles of combined concepts (curved beam and branch beam in the proposed design). Further, effort should be put into understanding the possible nonlinear behaviour of ASBBH under the influence of magnets or mechanical stoppers, aiming to reduce the anti-resonance valleys between obtained natural frequencies.

## Figures and Tables

**Table 1:** Material properties of the aluminium beam, Proof mass and MFC.

Parameters	Aluminium Beam	Proof Mass
Materials	Aerospace-graded Aluminium 2024	Steel
Elastic modulus (GPa)	70	210
Poisson’s ratio	0.33	0.28
Density (kg/m^3^)	2780	7900

**Table 2:** Material properties of MFC transducer.

Property	Young’s Modulus E1 (GPa)	Young’s Modulus E2 (GPa)	Poisson’s Ratio V12	Shear Modulus G12 (GPa)	Piezoelectric Charge Constants d33 (pC/N)	Piezoelectric Charge Constants d31 (pC/N)	Active Area Density (kg/m^3^)
Value	30.336	15.857	0.31	5.515	4.6 × 10^2^	−2.1 × 10^2^	5440

**Table 3:** Natural frequencies achieved by the three harvesters under FEA modal analysis.

Prototype	NF1	NF2	NF3	NF4	NF5	NF6
CBH	4.6351	65.083	67.488	176.37	177.19	322.04
SCBH	2.1566	4.3539	15.729	24.140	36.280	82.220
ASBBH	1.4358	2.2514	2.8898	3.6123	5.5713	6.8565

**Table 5:** Table of natural frequencies obtained in FEA study and experimental study.

Type of Analysis	NF1	NF2	NF3	NF4	NF5	NF6
ASBBH (FEA Study)	1.44	2.25	2.88	3.61	5.57	6.86
ASBBH (Experimental Study)	1.50	2.25	3.00	3.75	4.25	5.75

**Table 6:** Performance comparison of the ASBBH with existing designs.

Reference	Design Descriptions of the PEH	Types of Piezoelectric Materials	Frequency	Motion Type	Number of Natural Frequencies (NFs) < 10 Hz	Average Output Power	Remarks
Izadgoshasb, 2019 [27]	PEH with a double-pendulum system	MFC (M2814-P2)	1 Hz	Walking	1 NF	36 μW	Total weight of the system ≈ 250 g
Jiang, 2021 [66]	V-shaped bent beam harvester	PZT-5H	1 Hz	Feet stamping	None	100 μW	Volume ≈ 8400 mm^3^
Nastro, 2022 [67]	Wearable Ball-Impact PEH	PZT (RS-pro-285–784)	4 Hz	Wrist rotations	N/A	9.65 μW	Volume ≈ 8100 mm^3^
Piyarathna, 2023 [32]	Bent branched beam PEH	MFC (M2814-P2)	1 Hz	Walking	3 NFs	168 μW	Volume ≈ 8000 mm^3^
Proposed ASBBH	Arc shaped branch beam harvester	MFC (M2814-P2)	1 Hz	Walking	6 NFs	477 μW	Volume ≈ 5100 mm^3^

## Data Availability

Data could be provided upon request to the corresponding author.

## Supplementary Materials

The following supporting information can be downloaded at: https://www.mdpi.com/article/10.3390/s23115257/s1, Table S1: Parametric study of ASBBH with varying length.
