# Supplementary material for: Linear Segmented Arc-Shaped Piezoelectric Branch Beam Energy Harvester for Ultra-Low Frequency Vibrations
Source: Sensors (Basel). 2023 Jun 1;23(11):5257. doi: 10.3390/s23115257 (PMC10256088; doi:10.3390/s23115257)
Supplement: Supplementary file 1 [file sensors-23-05257-s001.zip › sensors-2364942-supplementary.pdf]

Supporting Information

Table S1 Parametric study of ASBBH with varying length

Design	Arch Length (mm)	Radius of Arch (R)(mm)	A (R-10-5)* (mm)	L1 (mm)	L2 (mm)	L3 (mm)	L4 (mm)	NF1 (Hz)	NF2 (Hz)	NF3 (Hz)
1	70	45	30	70	35	35	35	7.398	10.9531	11.588
2	70	45	30	70	70	35	35	5.8578	8.3974	10.100
3	70	45	30	70	70	70	35	5.6011	7.5171	8.2291
4	120	75	60	120	60	60	60	2.1357	3.0267	3.1129
5	120	75	60	120	120	60	60	1.4358	2.2514	2.8898
6	120	75	60	120	120	120	60	1.4164	2.2154	2.8296

*A = (Radius – Clamped Edge – length of the joint)
